# Individual Faces Were Not Discarded During Extracting Mean Emotion Representations

**DOI:** 10.3389/fpsyg.2021.713212

**Published:** 2021-10-04

**Authors:** Huiyun Li, Luyan Ji, Qitian Li, Wenfeng Chen

**Affiliations:** ^1^School of Psychology, Beijing Sport University, Beijing, China; ^2^State Key Laboratory of Brain and Cognitive Science, Institute of Psychology, Chinese Academy of Sciences, Beijing, China; ^3^Department of Psychology, University of Chinese Academy of Sciences, Beijing, China; ^4^Center for Brain and Cognitive Sciences, Department of Psychology, Faculty of Education, Guangzhou University, Guangzhou, China; ^5^Department of Psychology, Renmin University of China, Beijing, China

**Keywords:** ensemble representations, mean representations, individual representations, facial expression, attentional resources

## Abstract

Individuals can perceive the mean emotion or mean identity of a group of faces. It has been considered that individual representations are discarded when extracting a mean representation; for example, the “element-independent assumption” asserts that the extraction of a mean representation does not depend on recognizing or remembering individual items. The “element-dependent assumption” proposes that the extraction of a mean representation is closely connected to the processing of individual items. The processing mechanism of mean representations and individual representations remains unclear. The present study used a classic member-identification paradigm and manipulated the exposure time and set size to investigate the effect of attentional resources allocated to individual faces on the processing of both the mean emotion representation and individual representations in a set and the relationship between the two types of representations. The results showed that while the precision of individual representations was affected by attentional resources, the precision of the mean emotion representation did not change with it. Our results indicate that two different pathways may exist for extracting a mean emotion representation and individual representations and that the extraction of a mean emotion representation may have higher priority. Moreover, we found that individual faces in a group could be processed to a certain extent even under extremely short exposure time and that the precision of individual representations was relatively poor but individual representations were not discarded.

## Introduction

Humans are sensitive to ensemble representations of a group of objects (Albrecht and Scholl, 2010). An ensemble representation is any representation that is computed from multiple individual measurements, either by collapsing across them or by combining them across space and/or time (Alvarez, 2011). Extracting ensemble representations from collections of items is a basic element of routine decision-making (Peterson and Beach, 1967) and a highly efficient means of processing groups of similar objects. For example, in a basketball game, spectators are seen cheering after a team scores, but the degree of happiness of any individual cannot be discriminated. This situation occurs because the capacity of our visual system is limited (Cowan, 2001); to overcome this severe capacity limitation, the human brain does not generate precise individual representations of the facial expressions of each spectator. Instead, the brain employs statistical rules to compress redundant information and extracts mean emotion information from the group via ensemble coding (Alvarez, 2011). Research has suggested that we can precisely extract mean representations from groups of faces, but individual representations cannot be extracted (Haberman and Whitney, 2007, 2009). However, it has also been suggested that individual representations become more precise as the processing time increases (Li et al., 2016). To explain this apparently contradictory phenomenon, this study attempted to elaborate the processing mechanisms of these two types of representations.

There are two viewpoints regarding this issue. The “element-independent assumption” asserts that the extraction of a mean representation does not depend on recognizing or remembering individual items in the group and that the identification of individual items is not a prerequisite for extracting a mean representation (Luo and Zhou, 2018; Whitney and Leib, 2018). Therefore, the precision of individual representations is poor. In addition, observing the facial expressions of each individual in a group in detail is inefficient. Research has suggested that people can detect changes in the overall emotion from multiple faces but cannot locate faces with expressions change (Haberman and Whitney, 2011). People with developmental prosopagnosia could not identify individual faces in groups but could extract mean identity and mean emotion information (Leib et al., 2012). This finding suggests that the mechanisms used in processing mean representations and individual representations may be different.

In contrast, the “element-dependent assumption” proposes that the extraction of a mean representation is closely related to the processing of individual items in a group (Haberman et al., 2015). For example, faces of which one was unaware in a group could affect the perception of the mean emotion (Fischer and Whitney, 2011), and the ability to extract the mean representation declined when the processing of individual items was impaired (Neumann et al., 2017). Recent studies have found that the precision of a mean emotion representation depends on the precision of individual items (Sun and Chong, 2020; Lee and Chong, 2021). This viewpoint suggests that the poor precision of individual representations can be attributed to the fact that individual information is discarded after calculation or assembly or is difficult to be identified due to large amounts of noise (Alvarez, 2011; Baek and Chong, 2020).

Both view points recognize the poor precision of individual representations. However, individual representations can be as precise as mean representations. Kramer et al. (2015) reported that the mean representation of a set of familiar faces was as accurate as the individual representations. To explain this finding, it was suggested that processing individual familiar faces requires fewer attentional resources. The extraction of a mean representation was demonstrated to be capacity limited (Ji et al., 2018) similar to processing individual items in working memory. Therefore, to explore the processing mechanisms underlying the two types of representations, researchers need to consider attentional resources; however, limited research investigated how attentional resources affect the processing mechanisms of the two types of representations. Li et al. (2016) changed the exposure time to adjust the attentional resources allocated to individual items and found that when a set was presented for 50 ms, individual representations were imprecise, while the mean emotion representation was more precise; when the exposure time was longer (2,000 ms), both types of representations were precise (Li et al., 2016). These findings suggest that the processing of the two types of representations may compete for resources.

To adjust the attentional resources allocated to individual items, some studies varied the set size and found that the precision of the mean representation was affected by the set size (Leib et al., 2014; Peng et al., 2019). This finding suggests that the extraction of a mean representation might depend on the processing of individual items. Neumann et al. (2017) adjusted both the exposure time and set size to investigate the relationship between the mean identity representation and individual representations. Their results showed that when the exposure time was shortened or the set size was increased, the precision of both types of representations declined. In particular, when the set was presented for 50 ms or the set contained eight faces, the precision of both types of representations was very poor. The authors claimed that the precision of both types of representations was mutually dependent. Considering that performances in extracting mean representations of features at the same level (e.g., facial emotion and facial identity) are highly correlated (Hubert-Wallander and Boynton, 2015), it is reasonable to consider the following question: do the processes of extracting mean and individual emotion information from multiple faces have a mechanism similar to that of identity information?

To address this issue, we conducted two experiments with a classic member-identification paradigm (Haberman and Whitney, 2009). The aim of the present study was to explore the processing mechanisms of mean emotion representations and individual representations in a set and their relationship. We manipulated the attentional resources allocated to individual items in groups of faces by changing the exposure time of the set and the number of faces in the set (set size). We hypothesized that if extracting a mean emotion representation depends on individual representations, reducing the attentional resources allocated to individual items would affect the precision of both individual representations and the mean emotion representation. The results would show that when the exposure time was shortened or the set size was increased, the precision of individual representations declined, the precision of the mean representation also declined, and the two types of representations appeared to have a covariant relationship. In contrast, if extracting the mean emotion representation does not depend on individual representations, when the precision of individual representations declined, the precision of the mean representation would not be affected.

## Experiment 1

### Participants

We used G^∗^Power 3.1 software to calculate the sample size. Under the premise of a statistical test power 1 – *β* = 0.8 and α = 0.05, the estimated minimum sample size was 28.6. We recruited 30 Chinese undergraduate students (17 males, age = 21.97 ± 2.23 years) from Beijing Forestry University and surrounding universities. All participants had normal or corrected-to-normal vision and provided informed consent. After participating in the experiment, the participants were paid a certain amount of money. This research was approved by the local Institutional Review Board. The procedures used in this study adhere to the tenets of the Declaration of Helsinki.

### Stimuli

We collected 51 Asian face images from BU-4DFE (Yin et al., 2008) and the Internet that contained neutral and angry expressions from the same person. Mouths were closed in all images. Using Adobe Photoshop to process the images, we removed the external characteristics of each face (i.e., ears, hair, and neck) and retained the internal characteristics (i.e., eyes, nose, mouth, and cheeks). We produced grayscale images with uniform physical attributes, such as the face size, brightness, and contrast (Bex and Makous, 2002). We additionally recruited 25 Chinese undergraduate students who did not participate in the formal experiments (11 males, age = 22.44 ± 4.26 years) to evaluate the emotional valence, intensity, and arousal of the faces. The participants first judged the expression of each face, such as fear, sadness, surprise, disdain, disgust, happiness, anger, and neutrality. Then, they evaluated the emotional intensity (1 = completely without emotion, 10 = extremely intense emotion) and arousal (1 = extremely calm, 10 = extremely excited) of the faces on a 10-point Likert scale. After the statistical analysis, we obtained the proportion of images evaluated as each type of expression and their mean emotional intensity and mean arousal. We finally selected two face images with neutral and angry expressions of the same female (emotional intensity, 5.38; arousal, 6.03) to serve as the experimental stimuli.

**FIGURE 1:**
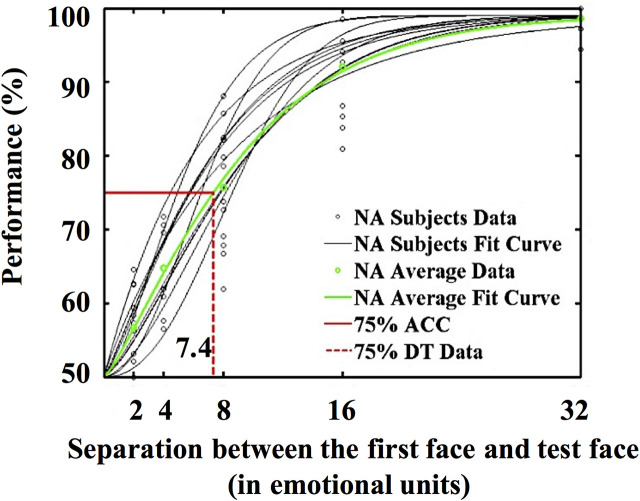
Function fitting results of the 75% discrimination threshold of the face sequence.

Following the morphing procedure used by Haberman and Whitney (2009), we generated a set of 50 faces by morphing between the neutral face and the angry face. Each face image was 120 × 151 pixels, and the visual angle was approximately 3.3° × 4°. The emotional expression of the faces ranged from neutral to angry, with Face 1 being neutral. The morphed faces were nominally separated from one another by emotional units (e.g., Face 2 was one emotional unit angrier than Face 1). To control variance in the face stimulus set, we further recruited 16 Chinese undergraduate students (four males, age = 22.5 ± 2.45 years) who did not participate in the formal experiments to measure the 75% discrimination threshold of the faces following the methods used by Haberman and Whitney (2007). The task was to judge whether an emotional face that appeared later was angrier than the previous one. The difference between the two faces was ±2/4/8/16/32 emotional units. This study involved 386 trials, including 10 practice trials and 376 formal trials. The results of the non-linear least squares fitting (MATLAB) indicated that the 75% discrimination threshold of the faces was 7.4 emotional units (see Figure 1).

### Apparatus and Procedures

The presentation of the stimuli and recording of the responses of the participants were controlled by a desktop computer. The experimental procedures were developed using E-prime 2.0. We used a 24” screen with a refresh rate of 100 Hz and resolution of 1,920 × 1,080 pixels. The participants were approximately 60 cm from the screen.

Faces were shown to the participants before the experiment. We randomly selected two images separated by 10 emotional units and asked the participants if they could distinguish the difference in the emotional intensity between the faces. The practice trials commenced after the participants confirmed that they could effectively discriminate the emotional intensity. At the start of each trial, a “+” fixation point was shown on the screen and displayed for 500 ms. Then, a set of four faces with different emotional intensities was presented. The mean emotional intensity of the face set in each trial was randomly designated. Two faces were more neutral and two faces were angrier than the mean emotional intensity. The difference between each two faces in the face set was at least 10 (larger than 7.4) emotional units, and the visual angle of the face set was approximately 7° × 8°. Each face image was 120 × 151 pixels, and the visual angle was approximately 3.3° × 4°. Then, a single test face was presented. The participants were instructed to indicate whether the test face was a member of the preceding face set. There were three types of test faces as follows: mean test faces, member test faces, and neither mean nor member test faces. The mean test faces included the mean emotional intensity face of each face set. The member test faces included member faces of the preceding face set. The neither test faces included faces that were five units away from the member faces in each face set. The full range of test faces varied from 20 units below the mean to 20 units above the mean. The test face remained on the screen until a response was received. Feedback was provided during the practice stage, but no feedback was provided during the formal experiment.

**FIGURE 2:**
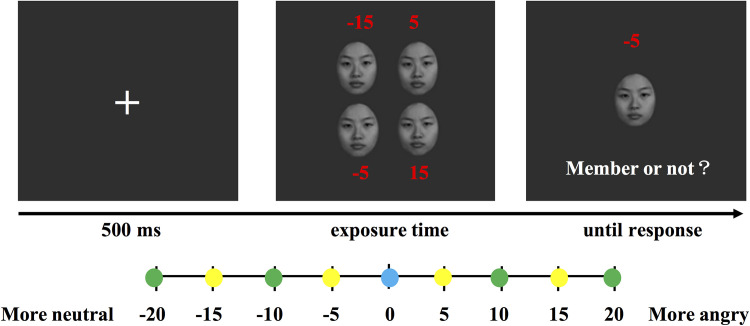
Task design in Experiment 1. The red numbers indicate the emotional intensity of each face compared to the mean face but were invisible in the trials. The test face could be any of the distances indicated by the circles. The yellow circles represent the member test faces of the face set. The green circles represent the neither test faces of the face set. The blue circle represents the mean test face of the face set.

According to the emotional intensity relationship between the face set and the test faces in the member identification paradigm, five sets of stimuli were chosen from the morphing image sequence. Each set contained nine face images, and the emotional intensity relationship between each type of face is shown at the bottom part of Figure 2. As test faces, nine face images appeared at the same times. One face image appeared only in one set. The exposure time of the face set was assigned using a block design, and the sequence was counterbalanced across the participants. Each block contained 196 trials, including 16 practice trials and 180 formal trials. The three types of test faces were randomized in each block. The experiment lasted approximately 30 min (see Figure 2).

### Data Analysis

Approximately 1% of the trials with response times less than 300 ms or longer than 3,000 ms for each participant were discarded. Following Haberman and Whitney (2009), the proportion of “yes” responses was analyzed. A “yes” response indicates that the participants believed that the test face was a member of the preceding face set. A Greenhouse-Geisser correction was applied when assumptions of sphericity were violated. A Bonferroni correction was used when multiple comparisons were performed.

### Results

**FIGURE 3:**
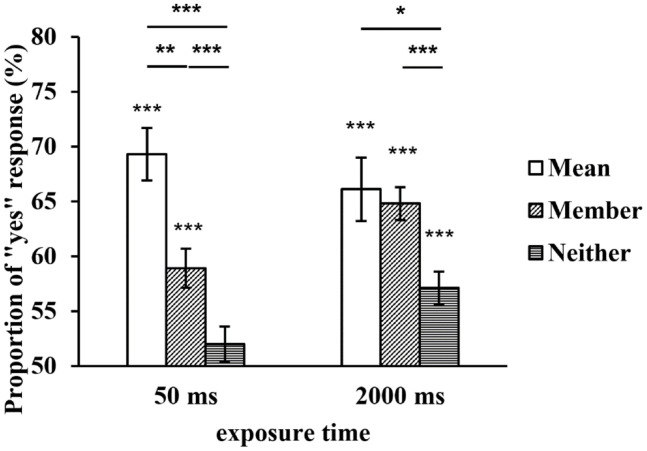
The proportion of “yes” responses to the test faces at two exposure times. *p < 0.05, **p < 0.01, ***p < 0.001. SEMs are shown.

A repeated-measures ANOVA was conducted with both the exposure time and type of test face as within-subject variables. The results showed that the main effect of the exposure time was not significant, *F*(1, 29) = 2.351, *p* = 0.136, *η*^2^_*p*_ = 0.075, but the main effect of the type of test face was significant, *F*(2, 58) = 21.551, *p* < 0.001, *η*^2^_*p*_ = 0.426. Further multiple comparisons revealed that the proportion of the mean test faces was significantly higher than that of the member test faces (*p* = 0.006), and the proportions of both were higher than that of the neither test faces (*ps* < 0.001). The interaction between the two factors was also significant, *F*(2, 58) = 4.144, *p* = 0.039, *η*^2^_*p*_ = 0.125. A simple effect analysis revealed that there was no significant difference in the proportion of the mean test faces between the two exposure times (*p* = 0.414), while the proportions of the member and neither test faces at 2,000 ms were significantly higher than those at 50 ms (*p* = 0.001, *p* = 0.007). In addition, when a set of faces was displayed for 50 ms, the proportion of the mean test faces was significantly higher than that of the member test faces (*p* = 0.002), and the proportions of both were higher than that of the neither test faces (*ps* ≤ 0.001). When a set of faces was displayed for 2,000 ms, there was no significant difference in the proportions of the mean and member test faces (*p* = 1), but the proportions of both were higher than that of the neither test faces (*p* = 0.04, *p* < 0.001) (see Figure 3).

To compare the proportion of “yes” responses to the test faces under each condition with chance level (50%), we conducted one-sample *t-*tests. The results revealed that when a set of faces was displayed for 50 ms, the proportion of the neither test faces did not significantly differ from chance level (*t*_*Neither*_ = 0.877, *p* = 0.388), but the proportions of the other two types of test faces significantly differed from chance level (*t*_*Mean*_ = 6.126, *p* < 0.001; *t*_*Member*_ = 4.497, *p* < 0.001). When a set of faces was displayed for 2,000 ms, the proportions of the three types of test faces significantly differed from chance level (*t*_*Mean*_ = 5.630, *p* < 0.001; *t*_*Member*_ = 9.876, *p* < 0.001; *t*_*Neither*_ = 3.614, *p* = 0.001) (see Figure 3).

### Discussion

The proportion of the neither test faces was not different from chance level when the set of faces was presented for 50 ms, indicating that the participants were not biased toward pressing “yes”. This proportion increased and rose above chance level as the set exposure time increased; this finding may be attributed to the participants giving more fixation time to the set and thus being able to perform more detailed processing of the member faces. Furthermore, the member faces contained more internal noise; thus, the neither test faces were prone to be misjudged as member faces. Our results indicated that the proportion of the member test faces was significantly higher than both chance level and that of the neither test faces regardless of the exposure time. This result is consistent with the findings reported by Li et al. (2016), but we found a new phenomenon in which the member faces in a set could be identified following a short exposure time, indicating that it is possible to extract individual representations to a certain degree even at an extremely short exposure time. Our results also indicated that the proportion of the mean test faces was significantly higher than both chance level and that of the neither test faces regardless of the exposure time. As previous studies found (Haberman and Whitney, 2009; Li et al., 2016), although the mean faces were not presented in sets, the participants tended to believe that these faces were previously presented. People can use implicit methods to quickly form a mean emotion representation of a set. However, in contrast to the results reported by Haberman and Whitney (2009), we found that the precision of the mean emotion representation was equally good regardless of the exposure time.

When a set of faces was presented for 2,000 ms, there was no significant difference between the mean and member test faces. The two types of representations were both precise. When the exposure time of the set was shortened, the precision of the individual representations declined, indicating that manipulating the set exposure time to change the attentional resources allocated to individual items in the set was effective. Nevertheless, the precision of the mean representation was not affected by the exposure time because the random internal noise of the member faces was cancelled during averaging (Alvarez, 2011; Baek and Chong, 2020). If the number of faces in a set is increased, would the mean representation still be unaffected by the exposure time? Would changes occur in the relationship between the two types of representations? In Experiment 2, we investigated the processing mechanisms of the two types of representations and their mutual relationship using an increased set size.

## Experiment 2

### Participants

We used G^∗^Power 3.1 software to calculate the sample size. Under the premise of a statistical test power 1 – *β* = 0.8 and α = 0.05, the estimated minimum sample size was 28.6. A new sample of 30 (12 males, age = 22.07 ± 1.97 years) Chinese undergraduate students from Beijing Forestry University and surrounding universities was recruited to participate in this experiment.

### Procedures

The set contained eight faces in each trial. Employing the methods described by Haberman and Whitney (2009), there were two instances of each face used in Experiment 1 in a set. The emotional intensity relationships among the four types of faces in a set were consistent with those described in Experiment 1. The faces were presented at random locations in a ring around the center of the screen, and the visual angle of the set was approximately 10° × 12°. The exposure time of a face set was assigned using a block design, and the sequence was counterbalanced across the participants. Each block contained 196 trials, including 16 practice trials and 180 formal trials. The three types of test faces were randomized in each block. At the end of the experiment, the participants were asked whether there were any faces with identical emotion among the eight faces. The other procedures were the same as those described in Experiment 1.

### Data Analysis

Approximately 2% of the trials with response times less than 300 ms or longer than 3,000 ms for each participant were discarded. A Greenhouse-Geisser correction was applied when assumptions of sphericity were violated. A Bonferroni correction was used when multiple comparisons were performed.

### Results

**FIGURE 4:**
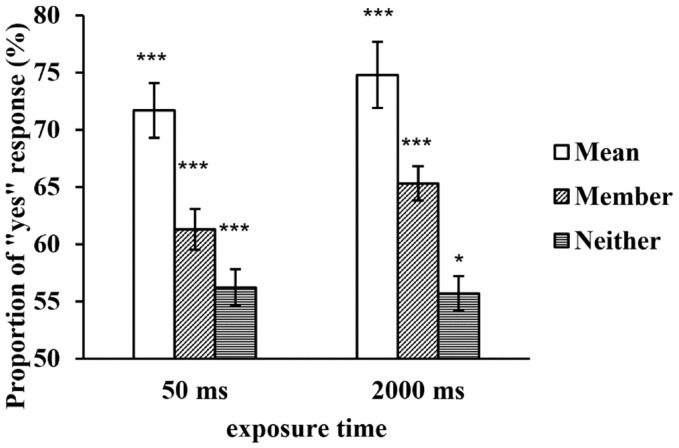
The proportion of “yes” responses to the test faces in a large set at two exposure times. *p < 0.05, **p < 0.01, ***p < 0.001. SEMs are shown.

A repeated-measures ANOVA found that the main effect of the exposure time was not significant, *F*(1, 29) = 1.632, *p* = 0.212, *η*^2^_*p*_ = 0.053, but the main effect of the type of test face was significant, *F*(2, 58) = 53.978, *p* < 0.001, *η*^2^_*p*_ = 0.651. Further multiple comparisons revealed that the proportion of the mean test faces was significantly higher than that of the member test faces (*p* < 0.001), and the proportions of both were higher than that of the neither test faces (*ps* < 0.001). The interaction between the two factors was not significant, *F*(2, 58) = 1.839, *p* = 0.168, *η*^2^_*p*_ = 0.060 (see Figure 4).

The one-sample *t-*tests revealed that when a set of faces was displayed for 50 ms, the proportions of the three types of test faces significantly differed from chance level (*t*_*Mean*_ = 10.387, *p* < 0.001; *t*_*Member*_ = 4.923, *p* < 0.001; *t*_*Neither*_ = 2.712, *p* = 0.011); when a set of faces was displayed for 2,000 ms, the proportions of the three types of test faces also significantly differed from chance level (*t*_*Mean*_ = 11.257, *p* < 0.001; *t*_*Member*_ = 8.481, *p* < 0.001; *t*_*Neither*_ = 2.4770, *p* = 0.01)(see Figure 4). In contrast to Experiment 1, the proportions of the neither test faces under both exposure time conditions significantly differed from chance level, which might be due to a “yes” response bias. However, this finding might also have been observed because processing member faces is accompanied by more internal noise when the set size is increased, resulting in the neither test faces being mistaken for member faces.

**FIGURE 5:**
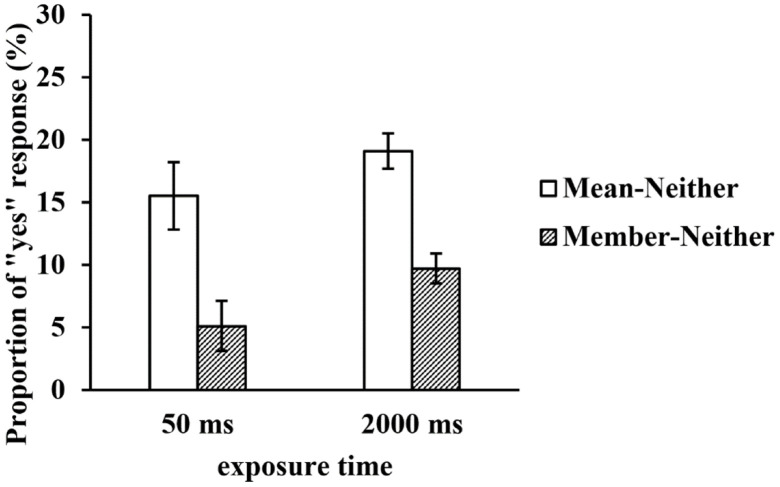
The net proportion of “yes” responses to the test faces in a large set at two exposure times.

To rule out a possible “yes” response bias, we subtracted the proportion of the neither test faces from those of the mean and member test faces under the two exposure time conditions, and we calculated the net proportion of “yes” responses to the mean and member test faces. A repeated-measures ANOVA of the net proportion revealed that the main effect of the exposure time was not significant, *F*(1, 29) = 3.693, *p* = 0.065, *η*^2^_*p*_ = 0.113, but the main effect of the type of test face was still significant, *F*(2, 58) = 33.645, *p* < 0.001, *η*^2^_*p*_ = 0.537. The net proportion of the mean test faces was significantly higher than that of the member test faces (*p* < 0.001). The interaction between the two factors was not significant, *F*(2, 58) = 0.146, *p* = 0.705, *η*^2^_*p*_ = 0.005 (see Figure 5).

### Discussion

Our results indicated that the proportion of the member test faces was significantly higher than both chance level and that of the neither test faces regardless of the exposure time. This result suggested that when the set size was large, the participants could extract individual representations even when the exposure time was shortened to 50 ms. However, in contrast to Experiment 1, the member test faces were not affected by the exposure time, and when a set of faces was displayed for 2,000 ms, the proportion of the member test faces did not increase. We propose that individual faces contain complex information and that processing multiple faces requires large amounts of attentional resources, but visual short-term memory is limited (approximately four items) (Luck and Vogel, 1997; Cowan, 2001). When a set is displayed for 50 ms, it may reach the limit for processing faces. Consequently, even an increase in the exposure time may not result in improving the precision of individual representations. Prior studies investigating the extraction of a mean representation from sets of dots with different diameters found that the maximum number of dots that could be processed was approximately four (Allik et al., 2013; Gorea et al., 2014). Because face information is more complex than dots, the number of faces that can be involved in processing may be even fewer.

The proportion of the mean test faces was significantly higher than both chance level and that of the neither test faces regardless of the exposure time, indicating that the mean emotion representation was also extracted when the set size was large. In contrast to Experiment 1, the proportion of the mean test faces was higher than that of the member test faces regardless of the exposure time. After ruling out the possibility that the participants were biased toward a “yes” response, our results indicated that this phenomenon still existed.

### Comparison of Experiments 1 and 2

To investigate the effect of the set size on the processing of the two types of representations at different exposure times, we analyzed the data by combining the two experiments and included the set size as a between-subject variable. A 2 (set size) × 2 (exposure time) × 3 (type of test face) repeated-measures ANOVA revealed that the main effect of the exposure time was marginally significant, *F*(1, 58) = 3.945, *p* = 0.052, *η*^2^_*p*_ = 0.064. The proportion when a set was displayed for 50 ms was lower than that when a set was displayed for 2,000 ms (*p* = 0.052). The main effect of the type of test face was significant, *F*(2, 116) = 68.024, *p* < 0.001, *η*^2^_*p*_ = 0.540. Further multiple comparisons revealed that the proportion of the mean test faces was significantly higher than that of the member test faces (*p* < 0.001), and the proportions of both were higher than that of the neither test faces (*ps* < 0.001). The interaction among the set size, exposure time, and type of test face was also significant, *F*(2, 116) = 3.981, *p* = 0.037, *η*^2^_*p*_ = 0.064. A simple effect analysis revealed that the interaction between the set size and type of test face was not significant when a set was displayed for 50 ms, *F*(2, 116) = 0.193, *p* = 0.825, *η*^2^_*p*_ = 0.003, and the proportions of the three types of test faces did not significantly differ between the two set sizes (*ps* > 0.05). The interaction between the set size and type of test face was significant when a set was displayed for 2000 ms, *F*(2, 116) = 4.776, *p* = 0.022, *η*^2^_*p*_ = 0.076. The proportion of the mean test faces with a set of four faces was lower than that with a set of eight faces (*p* = 0.020), and there were no differences in the proportions of the other two type of test faces between the two set sizes (*ps* > 0.05). The other main effects and interactions were not significant, *Fs* < 1.

Our results revealed that the proportion of the member test faces was not affected by the set size regardless of the exposure time. We speculate that the extraction of individual representations is constrained by the capacity of visual short-term memory (Luck and Vogel, 1997; Cowan, 2001) and that participants identifying four faces or even fewer faces reach the limit. In contrast, the proportion of the mean test faces was not affected by the set size when a set of faces was displayed for 50 ms and increased with the set size when the exposure time was prolonged, which is consistent with the findings reported by Leib et al. (2014).

## General Discussion

In this study, we employed a classic member-identification paradigm and manipulated the attentional resources allocated to individual items in a set to investigate the processing mechanisms of mean and individual emotion representations and their relationship. Our results showed that the precision of individual representations decreased as the exposure time decreased with smaller set sizes, but that of the mean representation was not affected by the exposure time; when the set size was increased, the precision of the two types of representations was not affected by the exposure time. The precision of the two types of representations was not affected by the set size at a shorter exposure time; when the exposure time was longer, the precision of the individual representations was not affected by the set size, but that of the mean representation increased as the set size increased. Our results indicate that the precision of the two types of representations did not covary and that two different processing mechanisms may exist.

The relationship between the two types of representations observed in this study is consistent with the results reported by Li et al. (2016) but not consistent with the results reported by Neumann et al. (2017). This study and the study by Li et al. (2016) focused on the ensemble coding of facial expressions, while Neumann et al. (2017) focused on the ensemble coding of facial identities. Thus, the ensemble coding of facial emotion and identity information might involve different processes. Peng et al. (2019) demonstrated that when the number of faces in a set was increased from four to eight, the precision of both the mean and individual identity representations declined. Based on these previous findings, it seems that the ensemble coding of facial identity information could be unique. However, Leib et al. (2014) found that the precision of mean identity representations improved as the set size increased, which is inconsistent with the findings reported by Neumann et al. (2017) and Peng et al. (2019). We propose that the different results might be due to stimulus variance in a set. Neumann et al. (2017) and Peng et al. (2019) used more than 40 faces with different identities as the experimental material, while Leib et al. (2014) employed morphing stimuli created from three faces, and the present study used morphing stimuli created from two faces. Therefore, the number of faces used in the different studies may have caused different stimulus variances in a set. Ji and Pourtois (2018) demonstrated that the effect of the set size on the mean emotion representation was moderated by emotional variance. When the emotional variance in a set was low, the precision of the mean emotion representation was not affected by the set size, but when the emotional variance in a set was high, the precision of the mean emotion representation declined as the set size increased. Furthermore, stimulus variance in a set affected the number of faces involved in ensemble coding (Ji et al., 2020). Ji et al. (2018, 2020) provided direct evidence suggesting that stimulus variance in a set could affect ensemble coding. The extraction of a mean representation is a capacity-limited perceptual process (Ji et al., 2018), and stimulus variance in a set might be an important factor causing different relationships between the mean representation and individual representations in different studies. Although the present study controlled the stimulus variance in a set, we did not investigate the effect of stimulus variance on the processing of the mean emotion representation. Future studies could explore the similarities and differences in the ensemble coding of emotion and identity information from the perspective of stimulus variance. Combined with the results reported by Neumann et al. (2017), we speculate that although the processing of facial emotion information and identity information depends on different systems (Bruce and Young, 1986), the ensemble coding of the two types of information may involve similar processing mechanisms.

Our study revealed that the precision of mean emotion representations increased as the set size increased. However, Ji and Pourtois (2018) found that when the emotional variance in a set was low, the precision of mean emotion representations was not affected by the set size; in contrast, when the emotional variance in a set was high, the precision of the mean emotion representation declined as the set size increased. In addition to the stimulus variance in a set discussed above, we propose that the difference between these findings could be due to the complexity of the stimuli. Ji and Pourtois (2018) employed both male and female faces with different identities and different emotion intensities; therefore, the faces in their set had mixed gender, emotion, and identity information. People can quickly and implicitly form mean representations of sets (Haberman and Whitney, 2007, 2009) and simultaneously extract multiple mean representations from multiple sets (Chong and Treisman, 2005). Although Ji and Pourtois (2018) manipulated the variance in emotional intensity, the participants might have simultaneously created mean gender or mean identity representations from the set while extracting a mean emotion representation as required by the experiment. Their results might have experienced interference from the processing of mean gender or identity representations. Thus far, no studies explored the issue of interference during the ensemble coding of different facial features, and this issue is worthy of future investigation. In addition, recent research has demonstrated that culture can modulate the processing of ensemble representations. People from various cultures exhibit differences in extracting mean representations from faces of different ethnicities (Im et al., 2017b; Peng et al., 2020). Persons from different cultures show different cognitive styles as follows: Asians are more sensitive to overall information, while Westerners are more concerned about local information (Masuda and Nisbett, 2001; Nisbett and Miyamoto, 2005). The present study used Asian participants and Asian faces as stimuli, while Ji and Pourtois (2018) used Western participants and Western faces as stimuli. The differences between the two results might also be attributed to culture.

Li et al. (2016) found that a mean emotion representation could be extracted when a set was displayed for 50 ms, and we found that a mean emotion representation could be extracted regardless of the set size and exposure time. This result supports the conclusion that the extraction of a mean emotion representation is an implicit, rapid, and flexible process (Alvarez and Oliva, 2008; Haberman and Whitney, 2009). We also discovered that the precision of the mean emotion representation was greater than that of individual representations when a set was displayed for 50 ms; this result may indicate that mean representations have higher priority in ensemble coding. People process global information first in visual perception. Navon (1977) used the global/local paradigm to prove that global attributes had priority over local attributes in visual object processing, which is called the global precedence effect. Giving priority to global attributes helps control the whole and further guide the analysis of local details of stimuli. Mean representations are a type of ensemble representations used to obtain overall information from complex stimuli; thus, such representations possibly have higher priority in visual perception. This view needs to be further tested by combining event-related potential, functional magnetic resonance imaging, and other techniques in the future.

In contrast to prior studies (Haberman and Whitney, 2009; Li et al., 2016), we found that individual representations could be processed to a certain degree when a set was presented for 50 ms. We speculate that the individual items received more processing resources after strictly controlling for the emotional variance in the set; therefore, the precision of individual representations increased. Previous research concluded that the poor precision of individual representations is due to individual information being discarded after calculation or assembly or being difficult to be identified due to the large amounts of noise (Alvarez, 2011; Baek and Chong, 2020). The present study verified that individual representations were not discarded after the extraction of mean representations. Hochstein and Ahissar (2002) and Hochstein et al. (2015) claimed that mean representations received higher priority in processing during the initial stage of information processing, while detailed individual information was subsequently processed. However, different brain mechanisms might be involved in the extraction of the two types of representations (Im et al., 2017a). We propose that two different pathways may exist for extracting mean representations and individual representations. Mean representations possibly receive higher priority in processing. Individual representations simultaneously receive a certain degree of processing that is easily affected by attentional resources.

Prior studies have claimed that processing a small number of individuals in a set could yield a mean representation (Myczek and Simons, 2008; Ji et al., 2020). We believe that there is insufficient time for subsampling when the exposure time of a set is extremely short and that even if a subsampling processing strategy was employed, the selection of a small sample in place of the set’s mean emotion would result in a large bias under the constraint of extremely short time. However, we found that the precision of the mean representation was not affected by the exposure time, and it was not affected by the set size even if the exposure time was extremely short. This finding supports the idea that the extraction of mean representations does not employ a subsampling strategy (Chong et al., 2008; Baek and Chong, 2020).

## Conclusion

The current study showed that while the precision of individual representations was affected by attentional resources, the precision of mean emotion representations did not change with it. Two different pathways may exist for extracting mean representations and individual representations. These results support the idea that extracting mean emotion representations possibly receive higher priority in processing. We also proved that individual items could be processed to a certain extent even in the case of a very short exposure time. Although the precision of the individual representations was relatively poor, individual representations were not discarded.

## Data Availability Statement

The original contributions presented in the study are included in the article/supplementary material, further inquiries can be directed to the corresponding author.

## Ethics Statement

The studies involving human participants were reviewed and approved by Department of Psychology, University of Chinese Academy of Sciences. The patients/participants provided their written informed consent to participate in this study. Written informed consent was obtained from the individual(s) for the publication of any potentially identifiable images or data included in this article.

## Author Contributions

HL designed this study and wrote the draft. QL and HL collected and analyzed data. HL, LJ, and WC interpreted data. LJ, QL, and WC revised the manuscript. All authors contributed to the article and approved the submitted version.

## Conflict of Interest

The authors declare that the research was conducted in the absence of any commercial or financial relationships that could be construed as a potential conflict of interest.

## Publisher’s Note

All claims expressed in this article are solely those of the authors and do not necessarily represent those of their affiliated organizations, or those of the publisher, the editors and the reviewers. Any product that may be evaluated in this article, or claim that may be made by its manufacturer, is not guaranteed or endorsed by the publisher.
